# Golgi-associated Rab GTPases implicated in autophagy

**DOI:** 10.1186/s13578-021-00543-2

**Published:** 2021-02-08

**Authors:** Qingchun Lu, Po-Shun Wang, Ling Yang

**Affiliations:** Department of Medical Genetics and Molecular Biochemistry, Lewis Katz School of Medicine at Temple University, 3440 N Broad St, Kresge Hall, Rm. 624, Philadelphia, PA19140 USA

**Keywords:** Autophagy, Rab GTPase, Golgi, Vesicle trafficking

## Abstract

Autophagy is a conserved cellular degradation process in eukaryotes that facilitates the recycling and reutilization of damaged organelles and compartments. It plays a pivotal role in cellular homeostasis, pathophysiological processes, and diverse diseases in humans. Autophagy involves dynamic crosstalk between different stages associated with intracellular vesicle trafficking. Golgi apparatus is the central organelle involved in intracellular vesicle trafficking where Golgi-associated Rab GTPases function as important mediators. This review focuses on the recent findings that highlight Golgi-associated Rab GTPases as master regulators of autophagic flux. The scope for future research in elucidating the role and mechanism of Golgi-associated Rab GTPases in autophagy and autophagy-related diseases is discussed further.

## Background

### Autophagy and its machinery

Autophagy is a cellular degradative pathway involving the delivery of cytoplasmic cargo to lysosomes and is highly conserved among eukaryotes ranging from yeast to mammalian cells [1–3]. To date, at least 30 different autophagy-related genes (Atg) have been identified to be associated with the regulation and execution of autophagy in yeast [4, 5]. The autophagic process comprises four major steps that are executed by specific *Atg* genes [6–8]. The first step of autophagy induction involves the formation (vesicle nucleation) and expansion (vesicle elongation and regulation) of an isolation membrane known as phagophore. Several distinct signaling pathways and proteins are involved in the execution of the initial step. However, it remains to be elucidated whether these signaling pathways work in concert or independently. The mammalian target of rapamycin (mTOR) kinase, a major regulator of cap-dependent translation in cells, is known to strongly inhibit autophagy. Availability of nutrients stimulates the binding of mTOR and ULK1-Atg13-FIP200 to form complexes on autophagic isolation membranes, thereby suppressing the phagophore expansion. Nutrient deprivation or rapamycin inhibits the association of mTOR, ULK1, and mTOR kinase activity, leading to the upregulation of autophagy in both yeast and mammalian cells [9, 10]. In addition, several protein regulators such as p53, c-jun-N-terminal kinase 1 (JNK1), eukaryotic initiation factor 2α (eIF2α), GTPases, and intracellular calcium are known to play an important role in regulating this initial step of autophagy [11–16]. These molecules and/or signaling pathways promote autophagy by activating the class III phosphatidylinositol 3-kinase (PI3K) Vps34 that initiates the formation of phosphatidylinositol 3-phosphate (PIP3) on lipids. The activity of Vps34 in promoting autophagy depends on its interactions within a multi-protein complex including the essential mammalian autophagy protein beclin 1 (BECN1, homolog of yeast Atg6) [17]. Additional proteins, namely, Bcl-2, Ambra1, Bif-1, and Atg14L, interact with Vps34 and beclin 1 to form functionally distinct multi-protein complexes that regulate different stages of autophagosome development and vesicular trafficking [18, 19]. The drug 3-methyladenine (3-MA), an inhibitor of class III PI3K, is frequently used for the pharmacological inhibition of autophagy in cells [20]. Thus, both rapamycin and 3-MA exert a significant influence on autophagy and the associated cellular pathways and processes.

The second step of autophagy begins with elongation followed by closure of the phagophore to form an autophagosome. Autophagosome is a double-layered vesicle that sequesters the cytoplasmic material using two separated but evolutionarily conserved ubiquitin-like conjugation systems [7, 8, 21]. In the first system, the E1- and the E2-like enzymes, Atg7 and Atg10, respectively, promotes the covalent association between Atg12 and Atg5. Subsequently, Atg16 associates with this complex to form an Atg5-Atg12-Atg16 heterotrimeric complex that associates primarily on the outer membrane of the growing autophagosome, hypothesized to mediate the curvature of the growing membrane [22, 23]. The second ubiquitin-like conjugation system results in the cleavage of the C-terminal of the microtubule-associated protein light chain 3 (LC3, a homolog of yeast Atg8) by Atg7 and the cysteine protease Atg4. Following cleavage, the E2-like enzyme Atg3 adds phosphatidylethanolamine (PE) to a conserved glycine residue present at the C-terminus of the cleaved LC3 (designated LC3-I) to form the well-known LC3-II or LC3-PE. In general, LC3 is soluble and dispersed throughout the cytoplasm, but upon cleavage and lipidation with PE, it is recruited to the outer and inner membrane of the growing autophagosome. LC3 is the only known protein that stably associates with the completed autophagosomes and is commonly used as a marker for studying autophagy. Autophagosome formation can be assessed biochemically by the ratio of lipidated (LC3-II) and non-lipidated (LC3-I) forms of LC3 [24, 25]. Once the matured autophagosome is formed, Atg9 and Atg18 act to remove and recycle Atg16, Atg12, Atg5, and outer membrane LC3 [26].

The third step of autophagy begins with the docking and fusion of mature autophagosomes with lysosomes to form autolysosomes. The engulfed material together with the inner membrane are degraded inside the autolysosome [7, 8, 27]. Lysosomal-associated membrane protein 1 and 2 (LAMP1 and LAMP2) and the GTP-binding protein Rab7 are involved in this process [28]. In addition, autophagosomes are capable of fusing with endosomes to form organelle amphisomes [29]. It remains to be elucidated whether amphisomes are distinct entities or are precursors for the formation of autolysosomes. The final step of autophagy is vesicular breakdown and degradation of cytoplasm-derived contents. Fusion of autophagosome and lysosome provide access to lysosomal proteases (such as cathepsin B, D, and L) and diverse hydrolytic enzymes to enter the autophagosome resulting in the degradation of the constituents of the inner autophagosomal membrane. Subsequent to degradation, the amino acids and lipids are exported from the autolysosome into the cytoplasm where they are recycled to generate new macromolecules [30].

### Role of Rab proteins in vesicle transport

**Fig. 1:**
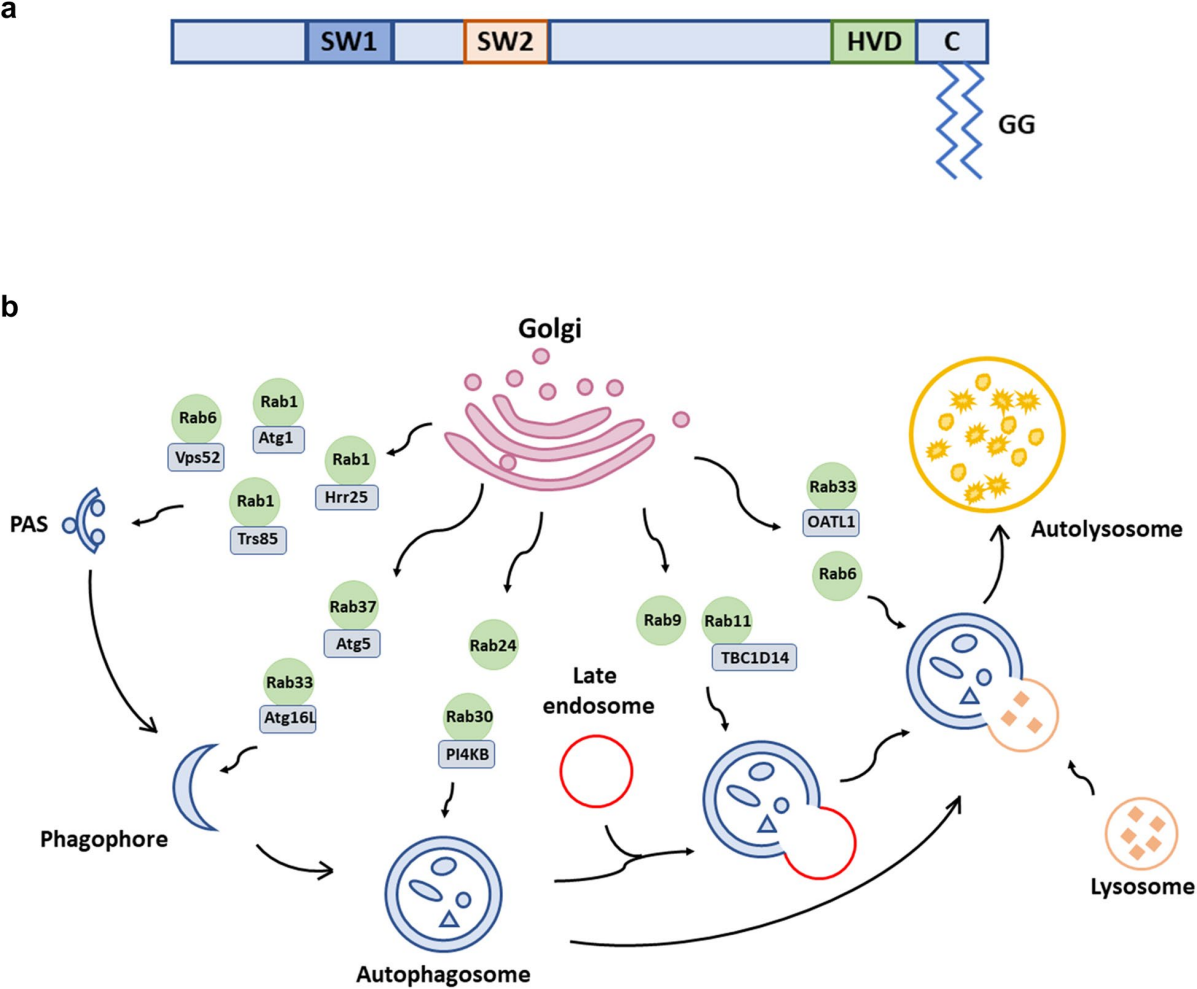
Representative structure of Rab GTPases and schematic model of the role played by Golgi-associated Rab GTPases with established functions in autophagic pathways. **a** The typical structure of a Rab GTPase. SW: switch regions. HVD: hypervariable domain. C: C-terminal prenylation motif. GG: geranylgeranyl groups. **b** Golgi-associated Rab GTPase and their binding partners in autophagy. Rab1 and Rab6 have crucial roles in PAS formation; Rab33 and Rab37 are participated in Phagophore formation; Rab24 and Rab30 are involved in autophagosome formation; Rab9 and Rab11 mediate autophagosome-endosome fusion; Rab6 and Rab33 mediate autophagosome-lysosome fusion. PAS, pre-autophagosomal structure

Rab proteins comprise a large family of small guanosine triphosphate (GTP)-binding proteins that play a crucial role in the regulation of intracellular vesicle trafficking [31–33]. The structure of Rab proteins is highly conserved across the family and consists of two switch regions, one hypervariable domain, and one C-terminal prenylation motif [34, 35]. The prenylation motif is responsible for modification of the geranylgeranyl group (GG) essential for the attachment of the Rab protein to the membrane (Fig. 1a). In mammalian cells, distinct Rab proteins localize at specific membrane-bound compartments or endocytic organelles where they modulate several cellular processes related to cytoplasmic cargo sorting, vesicle budding, docking, fusion, and membrane tabulation. In addition, they facilitate cytoskeletal translocation owing to their repeated alteration between an inactive guanosine diphosphate (GDP)-bound form and an active GTP-bound form [36]. The guanine nucleotide exchange factor (GEF) can essentially shift a Rab GTPase from the GDP-bound to the GTP-bound conformation, whereas the GTPase activating domain (GAP) exerts the opposite effect of inactivating GTPase [37, 38]. Importantly, several studies have shown that specific amino acid mutations could affect the switch from the GDP-bound to the GTP-bound conformation, and vice versa [39]. Therefore, an active GTP-bound form can further interact with its effectors or binding partners to fulfill the functions and mediate intracellular signals in response to physiological and metabolic demands.

Among the ~ 70 known Rab proteins, more than 20 are associated primarily with the Golgi apparatus and are considered Golgi-associated Rab GTPases [40]. Golgi is a highly dynamic organelle that plays a central role in the events related to vesicle transport. Vesicle organization and trafficking associated with Golgi relies on the Golgi-associated Rab GTPases [41]. Previous studies have demonstrated the role of Golgi-associated Rab proteins in regulating the formation of autophagosomes indicating its importance in autophagy [42–44]. A summative review covering the functions and mechanistic role of Golgi-associated Rab GTPases in autophagic flux would contribute to improved understanding of autophagy. Therefore, the present review is aimed at summarizing the existing knowledge related to the role of Golgi-related Rab GTPases and their signaling partners in autophagy (Fig. 1b).

## Main text

### Rab1

Rab1 GTPase (22 kDa) localized in the ER-Golgi intermediate is involved in the regulation of membrane trafficking from the ER to the Golgi [45, 46]. Currently, two isoforms of Rab1 GTPase have been identified, namely, Rab1A and Rab1B. They are highly conserved proteins with similar biochemical properties and functions [30, 47, 48]. Rab1 is an important regulator of several signaling pathways, including the mTOR pathway [49]. Overexpression of Rab1A drives the mTORC1 signaling and mTORC1-dependent growth in tumor by modulating the direct interaction between mTORC1 and Rheb. The biological activity of Rab1 is dependent on its GTPase activity, whereas the dominant-negative mutant Rab1aS25N and the nucleotide-free mutant Rab1aN124I perturb transport processes leading to the disruption of the Golgi apparatus [50, 51]. Similar results were reported to be associated with the overexpression of the dominant-negative mutant Rab1bN121I, which contributes to the disarrangement of the Golgi structure followed by the release of β-COP into the cytosol [52]. In addition, Rab1 binds directly to Golgi-84, GM130, and p115, all of which are required for the generation and maintenance of the Golgi structure [53]. Interestingly, Rab1b knockdown or overexpression of nucleotide-free mutant Rab1bN121I in CHO cells robustly suppressed autophagosome formation, suggesting that Rab1b-dependent vesicular transport from the ER is crucial for the initiation of autophagy [54]. The results were supported by the finding of Meiling-Wesse K et al., who found that Rab1 GEF Trs85, a component of the TRAPP complex, is essential for the biogenesis of pre-autophagosomal structure (PAS) [55]. Activated Ypt1/Rab1 recruits Atg1 kinase to PAS, bringing it into close proximity with the binding partner, Atg17. In addition, Ypt1/Rab1 binds directly to and spatially activates HRR25/casein kinase 1 delta (CK1δ), which in turn regulates vesicle transport and autophagosome formation [56, 57]. These results indicate the role of Rab1 as a pivotal regulator controlling the crosstalk between ER-Golgi traffic and autophagy.

### Rab6

Rab6 GTPase (24 kDa) with its intra-Golgi localization, serves as a trans-Golgi marker with a well-established role in retrograde transport within the Golgi and between the Golgi and ER or endosome membrane [58–60]. Rab6 binds to Golgi resident proteins such as the coiled-coil homodimer Bicaudal-D (BicD), which is necessary for tethering vesicles and Golgi membranes to microtubules [61, 62]. In addition, Rab6 is involved in cell cycle regulation and apical-basal sorting [63]. The yeast Rab6 ortholog (Ypt6) plays a crucial role in sorting vacuolar hydrolases by regulating endosome-to-Golgi traffic and is essential for autophagy initiation. Rab6 binds directly to Vps52, a subunit of the Golgi-associated retrograde protein (GARP) complex. GARP facilitates the delivery of Atg9 to PAS under stress conditions [64, 65]. In addition, Ypt6 facilitates the recruitment of the GARP tethering complex to the Golgi ensuring the retrieval of lysosomal sorting receptors. Importantly, knockdown of Ypt6 or its GEF Ric1/Rgp1 leads to autophagy defects [66]. Recently, Ayala et al. reported a novel function of Rab6 as an important regulator of balance between mTOR signaling and autolysosome function [67]. Rab6 deficiency enhances the number of enlarged autophagic vesicles and suppresses lysosomal function, resulting in a partial inability to deliver lysosomal hydrolases to autolysosomes. Furthermore, loss of Rab6 expression leads to a significant reduction of cell size and inactivation of insulin-mTOR signaling because of mis-sorting and internalization of the insulin receptor. These findings suggest the importance of Rab6 in controlling the dynamic balance between the formation and turnover of autophagosomes under specific nutrient conditions to avoid autophagic stress.

### Rab9

Rab9 GTPase (23 kDa) localized in the late endosome plays an important role in vesicle transport from the late endosome to the trans-Golgi network (TGN) [68]. Rab9 interacts with RhoBTB3, which is required for Golgi apparatus morphology as well as vesicle trafficking processes [69]. Knockdown of Rab9 attenuates the formation and decreases the number of late endosomes found in the perinuclear region, suggesting its necessity for the maintenance of late endosomes [70]. In addition, Rab9 is required for Atg5- and Atg7-independent autophagic pathways. Mouse embryonic fibroblasts lacking Atg5 or Atg7 expression could trigger autophagic flux and induce LC3 lipidation under specific metabolic stress conditions. The formation of autophagosomes, followed by fusion with late endosomes or TGN-derived vesicles, appears to be modulated in a Rab9-dependent manner [71, 72]. Intriguingly, increased Rab9 localization to autolysosomes was found with the active form Rab9Q66L, but not with the dominant-negative mutant Rab9S21 [70]. Thus, Rab9-dependent autophagy provides an alternative pathway to respond to metabolic stress in the absence of essential autophagy-related genes.

### Rab11

Rab11 GTPase (25 kDa) localized in the TGN/post-Golgi vesicles interact with perinuclear recycling endosomes to regulate transferrin recycling in CHO or BHK cells [73–75]. Rab11 also localizes on multivesicular bodies (MVBs) in K562 cells, and its overexpression enhances the formation of large MVBs. Autophagy induction by starvation or rapamycin treatment robustly promotes the fusion of MVBs and autophagosomes. This is perturbed by calcium chelators or by the overexpression of the dominant-negative mutant Rab11S25N [76], suggesting the involvement of a calcium- and Rab11-dependent regulation. Rab GTPase activating proteins (RabGAPs) with a Tre-2/Bub2/Cdc16 (TBC) domain is associated with membrane-bound compartments and autophagy [77]. RabGAP TBC1D14 binds to ULK1 and Rab11 and is followed by the disruption of the recycling endosome traffic. TBC1D14 and Rab11 cooperate to regulate membrane transport from recycling endosomes to autophagosomes under starvation. Overexpression of TBC1D14 enhances tubulation of ULK1- and Rab11-positive recycling endosomes in a Rab11-dependent manner, perturbing autophagosome formation [78, 79]. Therefore, TBC1D14- and Rab11-dependent membrane transport from recycling endosomes plays a pivotal role in the regulation of starvation-induced autophagy.

### Rab24

Rab24 GTPase (24 kDa) localized in the ER/cis-Golgi and late endosomes participate in autophagy-related processes [80]. Rab24 relocates to autophagic vacuoles and localizes with LC3 under starvation. This process is facilitated by vinblastine treatment [36], suggesting that Rab24 is essential for autophagosome formation in response to starvation stress. In addition, the bacterial pathogen *Coxiella burnetii* survives and replicates in LC3-positive phagolysosome compartments, and Rab24 overexpression accelerate the formation of *Coxiella*-containing vacuoles after bacterial infection. Overexpression of the dominant-negative mutant Rab24S67L significantly decreased the number and size of phagolysosomal structures [81, 82], suggesting that Rab24 promotes the maturation of phagolysosomes for *Coxiella* replication. Furthermore, Rab24 contributes to the degradation of aggregated proteins in rat cardiomyocytes. Glucose deprivation induces oxidative stress and enhances the formation of aggregates and aggresomes of polyubiquitinated proteins that localize with endogenous Rab24 and LC3 [83].

### Rab30

Rab30 GTPase (23 kDa) localized in trans-Golgi and is commonly found in metazoans. Previous studies have revealed the interaction of Rab30 with a number of Golgi proteins in *Drosophila melanogaster*, including golgins dGCC88, dGolgin-97, dGolgin-245, dGM130, as well as the fly orthologs of the coiled-coil proteins p115 and BicD [84]. GCC88, GM130, and p115 modulate the Golgi architecture, while Golgin-97, Golgin-245, and BicD are crucial for vesicular trafficking [85–89]. In addition, Rab30 has been identified as a target of c-Jun N-terminal kinase (JNK) signaling and functions as a TGN-resident modulator of intracellular trafficking during Drosophila morphogenesis [90]. Importantly, Rab30 primarily localizes in the trans-Golgi region to facilitate the structural maintenance of its morphological integrity. However, inhibition of Rab30 does not affect the trafficking of anterograde or retrograde cargo through the secretory pathway [91]. Furthermore, Oda et al. identified Rab30 as a novel regulator of autophagy induction in Group A *Streptococcus* (GAS) infection. They found that Rab30 is recruited to GAS-containing autophagosome-like vacuoles (GcAVs) that are dependent on its GTPase activity. In addition, Rab30 is essential for the formation of GcAV, followed by GAS degradation in autolysosomes. Although starvation induces Rab30 localization in autophagosomes, it is dispensable for starvation-induced autophagosome formation [92]. Recently, Nakajima et al. revealed that Rab30 promotes the recruitment of phosphatidylinositol 4-kinase beta (PI4KB) to the Golgi and GcAVs. They revealed a direct interaction between RAB30 and PI4KB, in which knockdown of RAB30 inhibited the localization of PI4KB to the TGN and GcAVs, providing evidence for the coordinative functions of RAB30 and PI4KB with regard to the xenophagic machinery [93].

### Rab33

Rab33 GTPase (27 kDa) localized in the Golgi apparatus is involved in intra-Golgi transport. Currently, it is known to have two isoforms: Rab33A and Rab33B. Rab33A is specifically expressed in the brain and immune system, while Rab33B is widely expressed in mammalian tissues [94–96]. Rab33B is known to bind with its effector, Golgi protein GM130, which is essential for Golgi-ER retrograde transport [97, 98]. Rab33A and Rab33B interact with Atg16L, regulating the stability of the Atg12-Atg5-Atg16 conjugation system essential for autophagosome formation. Overexpression of the constitutively active Rab33Q92L induces LC3 lipidation even under nutrient-rich conditions [99]. In addition, Rab33B interacts directly with RabGAP OATL1 to regulate autophagosomal maturation and participates in the fusion of autophagosomes and lysosomes. Overexpression of Rab33Q92L perturbs the fusion of the autophagic flux due to OATL1 inactivating Rab33B activity, thus increasing lipidation of LC3 but no colocalization with lysosomal membrane protein LAMP1 [100], indicating that Rab33B-overexpressed cells have limited capability to complete the autophagic flux. Recent studies prove the interaction of Rab33A with Atg16L and the necessity of this interaction for dense-core vesicle localization of Atg16L in neuroendocrine PC12 cells. Knockdown of endogenous Atg16L in cells results in perturbation of hormone secretion, independent of autophagic activity [99, 101, 102].Thus, Atg16L may serve as a Rab33A effector in neuroendocrine cells to control autophagy and secretion from dense-core vesicles.

### Rab37

Rab37 GTPase (25 kDa) localized in the Golgi apparatus participates in autophagosome formation [103]. Rab37 is involved in various physiological and pathological processes, such as mast cell degranulation and insulin secretion by regulating exocytosis [104–106]. In addition, Rab37 functions as a tumor suppressor gene by suppressing cancer metastasis [107, 108]. Besides these functions, Rab37 is associated with autophagy. Previous studies have shown that upon starvation, Rab37 localizes to autophagosomes and interacts directly with ATG5, recruiting it to the isolation membrane. As a crucial protein required for the initiation of pre-autophagosome formation, ATG5 recruits ATG16L1 to promote the assembly of the ATG5-ATG12-ATG16L1 complex, thus, facilitating autophagosome genesis. The constitutive negative mutant RAB37T43N, the mutated form of RAB37 that does not bind with GTP is scarcely associated with ATG5. Thus, RAB37 regulates autophagy in a GTP-dependent manner [109].

## Conclusion and future perspectives

The involvement of Golgi-associated Rab GTPases in the initiation, formation, maturation, and fusion of autophagosome summarized in this review suggests the crucial role played by them in autophagy. Among the ~ 20 Golgi-associated Rab GTPases, eight have been shown to be associated with autophagy. The involvement of Golgi-associated Rab GTPases in autophagy has received increased attention. Rab1 and Rab6 are recruited to PAS and is necessary for autophagosome biogenesis [57, 64, 65]. Rab33 and Rab37 are participated in Phagophore formation [99, 109]. Rab24 and Rab30 are essential for autophagosome formation [81, 82, 92, 93]. Rab6 and Rab33 are crucial for autophagosome-lysosome fusion [100]. Rab9 and Rab11 play a pivotal role in the autophagosome-endosome fusion stage [71, 72, 78, 79]. However, a comprehensive understanding of Golgi-associated Rab GTPase-related membrane trafficking events regarding the optimization of autophagic flux remains limited.

Although studies elucidating the role and mechanism of Rab GTPases have greatly improved our understanding of them in the past years, the underlying mechanisms of these GTPases are complex and require further investigation. Previous studies have shown that Rab1 might regulate Atg19 by activating the kinase activity of Hrr25/CKIδ. In addition, the GEF TRAPPIII of Rab1 is essential for the trafficking of Atg9 vesicles in the Cvt pathway, implying diverse roles of Rab1 in autophagy [57]. Rab30 is essential for the morphological integrity of the Golgi complex. A previous proteomic screen revealed that Rab30 interacts with a large set of Golgi proteins (such as golgins and the GARP complex), implying its participation in autophagy and regulation of multiple stages of Golgi homeostasis [93]. Rab33 may regulate autophagy by forming a complex feedback loop with three other factors: the Atg12-5-16L1 complex, Atg8 homologs, and OATL1 [100]. In addition, Rab GTPases can fulfill their function through binding their Rab effectors, which are specific proteins that interact with the GTP-bound form of Rab GTPase [33, 110]. Some Rab effectors are involved in autophagy by interacting with autophagy proteins. For example, Rab1 effectors C9orf72 and SMCR8 regulate autophagy by facilitating ULK1 transport to phagophores [111–113], suggesting Rab GTPases may also control autophagy via their specific effectors. These findings imply the diverse functions and mechanism of Golgi-associated Rab GTPases. Thus, future intensive studies on the precise molecular mechanism of Golgi-associated Rab GTPases are required to fully understand their importance in autophagy.

Autophagy is important for maintaining cellular homeostasis and is involved in the pathogenesis of a myriad of diseases, such as metabolic disorders, cancers, neurodegenerative diseases, and pathogen infections [114–117]. Interestingly, alteration in Golgi-associated Rab GTPase is also implicated in these diseases. Rab9 and Rab11 are important for energy metabolism: Rab9 overexpression reduces lipid storage [118]; Rab11, by affecting insulin sensitivity, is involved in diabetes pathogenesis [119]; [120–122] Rab1, Rab6, Rab9, and Rab11 participate in cancer progression: Rab1 regulates the development of hepatocellular carcinoma by modulating the mTOR pathway [120]; Rab6 interact with myosin II and acts as a negative regulator in multiple cancer cells, including lung cancer and osteosarcoma [121]; the progression of gastric cancer can be suppressed by inhibiting the Akt pathway via Rab9 silencing [122]; and the overexpression of Rab11 indicates poor survival time in patients with colorectal carcinoma [123]. In addition, Rab1 and Rab11 play crucial roles in the development of the nervous system[124, 125]: Rab1 protects against neuron loss in an animal model of Parkinson’s disease [124]; decreased Rab11 is observed in multiple neurodegenerative diseases; and overexpression of Rab11 slows down the progress of these diseases [125]. Rab1, Rab6, Rab11, Rab30, and Rab33B are involved in pathogen infections: the *Legionella pneumophila* effector SetA modifies the GDP-bound form of Rab1, thus, regulating its activity [126]; Rab6 affects the proliferation of *Staphylococcus aureus* in macrophages [127]; Rab11 contributes to the assembly of the core of influenza A [128]; Rab30 is involved in the immune response to GAS infection [93]; and Rab33B participates in hepatitis B virus assembly by regulating its nucleocapsid formation and trafficking [129]. In summary, both Golgi-associated Rab GTPases and autophagy are involved in the pathogenesis of the same set of diseases. Therefore, it is important to determine whether the involvement of Golgi-associated Rab GTPases in the pathogenesis of these diseases is through regulation of autophagy. A comprehensive understanding of Golgi-associated Rab GTPases in autophagy provides novel scientific knowledge related to autophagy and the pathogenesis of autophagy-related diseases.

## Data Availability

Not applicable.

## Abbreviations

3-MA: 3-Methyladenine
BHK: Baby hamster kidney
BicD: Bicaudal D
CK1δ: Casein kinase 1 delta
DCV: Dense-core vesicle
eIF2α: Eukaryotic initiation factor 2α
ER: Endoplasmic reticulum
GARP: Golgi-associated retrograde protein
GAP: GTPase activating domain
GAS: Group A Streptococcus
GcAVs: GAS-containing autophagosome-like vacuoles
GDP: Guanosine diphosphate
GEF: Guanine nucleotide exchange factor
GG: Geranylgeranyl groups
GTP: Guanosine triphosphate
JNK: C-jun N-terminal kinase
LC3: Microtubule-associated protein light chain 3
LAMP1: Lysosomal-associated membrane protein 1
LAMP2: Lysosomal-associated membrane protein 2
MEFs: Mouse embryo fibroblasts
mTOR: Mammalian target of rapamycin
mTORC1: Mammalian target of rapamycin complex 1
MVB: Multivesicular bodies
PI3K: Phosphatidylinositol 3-kinase
PIP3: Phosphatidylinositol 3-phosphate
PE: Phosphatidylethanolamine
PAS: Pre-autophagosomal structure
PI4KB: Phosphatidylinositol 4-kinase beta
RabGAP: Rab GTPase activating protein
TGN: Trans-Golgi network
ULK1: Unc-51 Like Autophagy Activating Kinase 1
